# Network Model to Predict Age‐Related Transcriptional Reprogramming

**DOI:** 10.1111/acel.70683

**Published:** 2026-08-22

**Authors:** Tyler J. McNeill, Fabrisia Ambrosio, Hirotaka Iijima

**Affiliations:** ^1^ Discovery Center for Musculoskeletal Recovery Schoen Adams Research Institute at Spaulding Charlestown Massachusetts USA; ^2^ Department of Physical Medicine and Rehabilitation Spaulding Rehabilitation Hospital Charlestown Massachusetts USA; ^3^ John A. Paulson School of Engineering and Applied Sciences Harvard University Cambridge Massachusetts USA; ^4^ Department of Physical Medicine and Rehabilitation Harvard Medical School Boston Massachusetts USA

**Keywords:** cell communication, extracellular signaling, gene expression profiling, *in silico* simulation, osteoarthritis

## Abstract

Understanding how secreted factors from aged tissue, often referred to as the senescence‐associated secretome, reshape cellular phenotypes remains a major challenge due to the complexity of downstream molecular cascades. Here, we present a computational framework for *in silico* perturbation modeling designed to predict distinct transcriptional responses to age‐specific extracellular environmental cues. We exemplify applications of this framework using articular chondrocytes exposed to secretomes derived from infrapatellar fat pads—an integral component of the cartilage microenvironment—excised from the knee joints of young and aged animals. First, we accessed public transcriptomic data of cartilage from healthy and osteoarthritic knee joints and constructed a cartilage‐specific co‐expression network using topological overlap matrices, which measure network interconnectedness. We then implemented a Random Walk with Restart to simulate the downstream signal propagation of differentially expressed ligands secreted from young and aged infrapatellar fat pads. We benchmarked predicted perturbation signatures against RNA‐seq data from aged chondrocytes treated in vitro with either young or aged infrapatellar fat pad‐conditioned medium. Our evaluation pipeline included functional enrichment comparison and receiver operating characteristic analysis. These analyses confirmed that simulated perturbations recapitulated chondrocyte signaling pathways modulated by young and aged infrapatellar fat pad secretomes, including primary effects on mitochondrial respiration, a central hallmark of aging. The network paradigm introduced here provides a data‐driven strategy to disentangle how complex, age‐dependent extracellular environments influence cellular fate. Ultimately, we anticipate that this pipeline can be extended to diverse tissues and age‐related diseases to guide the development of interventions that restore youthful cellular phenotypes.

## Graphical abstract

This study presents a network‐based model to predict intracellular responses to shifts in the extracellular environment and demonstrates the utility of the framework by estimating chondrocyte responses to age‐associated extracellular changes. Extracellular ligands are mapped to corresponding receptors, which serve as seed nodes for signal propagation through a context‐specific gene network using the random walk with restart (RWR) algorithm. RWR generates a ranked list of genes, with top‐ranked genes grouped into functional modules to predict downstream signaling responses.

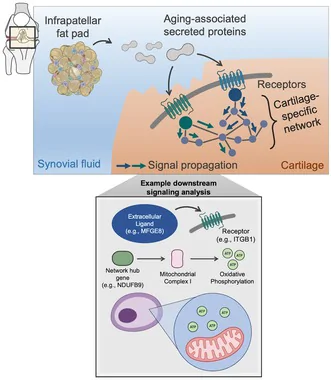

## 1 Introduction

Aging drives widespread molecular remodeling across tissues, leading to the disruption of cellular function and the gradual loss of tissue homeostasis (Rando and Jones 2021). Yet, experimentally identifying the causal mediators of aging and tracing their downstream effects remains a significant challenge—particularly in complex, low‐turnover tissues such as articular cartilage, where limited cell renewal and gradual changes make the spatial and temporal patterns of aging more difficult to map. Recent advances in omics‐based technologies have enabled comprehensive profiling of transcriptomic and proteomic alterations in aging tissues (Huang et al. 2025; Takasugi et al. 2024), including those within the knee joint (Liu et al. 2025). However, while differential expression analyses are becoming increasingly common in aging research, computational methods that dynamically simulate the propagation of extracellular perturbations through tissue‐specific molecular pathways remain underutilized. Such an integrative approach would offer a powerful tool to reveal downstream signaling mechanisms and predict tissue responses to external stimuli. Without broader integration of these computational pipelines into aging research, our capacity to uncover systemic drivers of age‐related pathophysiology and to develop targeted interventions for these diseases is severely limited.

Network propagation models, such as Random Walk with Restart (RWR), offer a mathematically rigorous approach to model the flow of information through biological networks (Lovász 1993). RWR models explore topological structures by simulating a particle that moves between nodes within a network. Beginning at a starting point designated as a seed node, the particle either moves to a random neighboring node or restarts at the seed node. As such, dependent on network topology, certain nodes will be visited more frequently than others, thereby allowing users to rank genes based on nodal proximity within the network (Valdeolivas et al. 2019). Thus far, these methods have been applied to prioritize disease‐associated genes (Qian et al. 2014), predict drug targets (Lee and Nam 2018), and reconstruct signaling cascades (Navlakha et al. 2012). Given that aging and aging‐associated diseases are characterized by not only intrinsic cellular dysfunction but also by altered paracrine and endocrine signaling (Guo et al. 2022), it is essential to integrate computational tools capable of modeling extracellular ligand inputs alongside intracellular transcriptional states in aging research. However, the use of these *in silico* strategies to explore mechanisms underlying cellular aging has thus far been limited.

In this study, we developed and validated an *in silico* perturbation framework that can be broadly applied to integrate secretome data derived from mass spectrometry‐based proteomics with tissue‐specific transcriptional networks to simulate receptor‐mediated downstream cellular responses. To demonstrate the utility and reliability of our approach, we applied this framework to cartilage aging by modeling chondrocyte responses to soluble factors in the microenvironment. By seeding perturbations from receptor nodes for ligands identified within secretome data and simulating signal diffusion using RWR, we generated ranked gene activity profiles to predict downstream responses. We evaluated this modeling output against experimental RNA‐seq data obtained from aged chondrocytes treated with media conditioned in the presence of either young or aged infrapatellar fat pads (IFP). Functional enrichment analysis and benchmarking confirmed that this approach recapitulates key biological processes observed in vitro. Our findings support the use of network‐based perturbation modeling as a systems‐level strategy for identifying candidate mediators of age‐related tissue dysfunction.

## 2 Materials and Methods

### 2.1 Preprocessing of RNA‐Sequencing Dataset of Human Articular Cartilage

We retrieved a publicly available RNA sequencing dataset, GSE114007, from the Gene Expression Omnibus (GEO) database. This dataset contains raw count data derived from human cartilage samples, including both healthy (*n* = 18) and knee osteoarthritis (KOA; *n* = 20) tissues (Fisch et al. 2018). All preprocessing steps were conducted using R studio with the Bioconductor packages. The dataset metadata was first obtained using the getGEO function from the GEOquery package. Raw gene‐level count matrices were downloaded from GEO Files S1 and S2 and imported separately for healthy and KOA cartilage. A DGEList object was constructed using the raw count matrix and the corresponding group annotations. Lowly expressed genes were filtered out using the filterByExpr function, which ensures sufficient expression across biological replicates. Library size normalization was performed using the trimmed mean of *M*‐values (TMM) method via the calcNormFactors function. Normalized expression values were calculated in counts per million (CPM), accounting for library size differences.

### 2.2 Construction of de Novo Gene Network via Weighted Gene Co‐Expression Network (WGCNA)

Using the WGCNA package (Langfelder and Horvath 2008), we built a weighted gene co‐expression network. The key parameter, *β*, for weighted network construction was optimized to maintain both the scale‐free topology and sufficient node connectivity, as recommended in the manual. The choice to enforce a scale‐free topology during the network construction was based on the biological relevance of this feature, as biological networks exhibit a scale‐free distribution (Albert 2005). This topology helps maintain key functional modules and ensures that the network structure aligns with known biological principles, thereby enhancing the interpretability of the results. A topological overlap matrix (TOM) was then formulated based on the adjacency values to calculate the corresponding dissimilarity (1‐TOM) values. To reduce network density while retaining biologically meaningful edges, we extracted the top 50,000 edges (ranked by TOM similarity) to form an adjacency list. Module identification was accomplished with the dynamic tree cut method by hierarchically clustering genes using 1‐TOM as the distance measure with a minimum size cutoff of 30 and a deep split value of 2 for the resulting dendrogram. A module preservation function was used to verify the stability of the identified modules by calculating module preservation and quality statistics in the WGCNA package (Langfelder and Horvath 2008). The filtered network was imported into the igraph package to construct a directed graph, with edge weights representing TOM similarity. All nodes were named with corresponding gene symbols. Duplicate and self‐loop edges were removed. The resulting network served as the substrate for signal diffusion modeling. In the current framework, neighboring nodes are defined as nodes connected by edges within the weighted gene co‐expression network. Network proximity is quantified by the stationary probability of visiting each node during repeated random walks with restart, thereby capturing both direct and indirect connectivity to the seed nodes within the global network.

### 2.3 IFP Sample Preparation and LC–MS/MS Mass Spectrometry‐Based Proteomics

To establish a source of extracellular signaling information to propagate through the cartilage‐specific gene–gene interaction network, we collected secretome data from IFPs derived from both young (4–6 months old) and aged (21–24 months old) male C57BL/6 mice. IFPs are an intraarticular adipose tissue that modulate chondrocyte health via paracrine signaling and influence the progression of KOA (D'Amico et al. 2025; Wang, Seale, and Furman 2024). Thus, the IFP represents an ideal source of extracellular signaling information for *in silico* perturbation of articular cartilage‐specific transcriptional networks. We have previously reported on the dissection of murine IFPs and subsequent secretome profiling used to identify perturbation inputs for the current network model (D'Amico et al. 2025). All animal procedures detailed in our previous publication were approved by the Institutional Animal Care and Use Committee of Massachusetts General Hospital (Protocol IDs: 2022N000171 and 2022N000148). Briefly, IFPs were harvested from young and aged mice based on previously established protocols (Futami et al. 2012) and washed in phosphate buffered saline prior to use. After washing, IFPs were cultured in 48‐well plates with 300 μL of chondrocyte proliferation media for 72 h before the conditioned medium was collected. Mass spectrometry (LC–MS/MS) was used to identify and quantify proteins in the secretome. Raw data were searched against the mouse UniProt database using Proteome Discoverer v2.3 and label‐free quantification was performed. Differentially abundant proteins were mapped to a curated secreted protein database in mice (Wang, Ren, et al. 2024), and therefore, were considered putative extracellular ligands.

### 2.4 Ligand‐Receptor Network Curation

Ligand‐receptor interactions were obtained from the publicly available NicheNet ligand‐receptor dataset (Browaeys et al. 2020), which integrates curated interactions from KEGG, Ramilowski et al. (Ramilowski et al. 2015), and the IUPHAR Guide to Pharmacology (via Harmonizome), as well as protein–protein interaction–based inferred ligand–receptor pairs. Ligands identified by mass spectrometry were cross‐referenced with the NicheNet ligand‐receptor database to extract matching receptor genes expressed in the cartilage transcriptome dataset. Low‐confidence and redundant interactions were excluded. The resulting ligand–receptor pairs formed the input for network propagation modeling. A complete list of the ligand‐receptor pairings associated with differentially expressed IFP‐secreted factors is found in Table S1.

### 2.5 In Silico Perturbation via Random Walk With Restart

To simulate the impact of these extracellular ligands on intracellular transcriptional responses, we employed the RWR algorithm implemented via the RandomWalkRestartMH package in R (Valdeolivas et al. 2019). RWR is a network propagation model that simulates the flow of information through a biological network by propagating signals starting from seed nodes—in this case, the receptor genes of secreted proteins identified from the mass spectrometry data. Receptor genes corresponding to each ligand were designated as seed nodes, and signal diffusion was simulated over the cartilage network with the restart probability set to the default (0.7), as established in prior literature (Li and Patra 2010; Smedley et al. 2014). The algorithm computes a steady‐state probability distribution for each node, reflecting its network proximity to the seed(s). The resulting RWR score matrix ranks all nodes (genes) according to their influence from each ligand–receptor seed.

### 2.6 Primary Murine Chondrocyte Isolation, Culturing, and RNA‐Sequencing

To validate the network model results, we benchmarked the *in silico* outputs against RNA‐sequencing data from aged chondrocytes treated with IFP‐conditioned media that we have previously reported (D'Amico et al. 2025). In that study, chondrocytes were isolated from aged male mice using previously established protocols (Gosset et al. 2008). To assess the biological impact of IFP‐secreted factors, the isolated murine chondrocytes were cultured for 1–2 weeks and then treated with media conditioned in the presence of either young or aged IFPs for 72 h.

Following treatment, four samples per group (~9 × 10^6^ chondrocytes/sample) were collected for RNA isolation using the Qiagen RNeasy kit, following the manufacturer's protocol with RNase/DNase‐free materials. Briefly, chondrocytes were trypsinized, pelleted, and lysed in buffer RLT. Lysates were homogenized by syringe shearing and processed through RNeasy spin columns. RNA was eluted in RNase‐free water and stored at −80°C prior to bulk RNA sequencing. Samples with Phred scores < 20 were excluded; all samples analyzed had scores > 30. Reads were aligned to the *Mus musculus* mm10 genome using STAR (v2.7.10b). Differentially expressed genes were defined as those with a false discovery rate < 0.05 and |log2 fold change| > 1 between chondrocytes exposed to young and aged IFP conditioned media.

### 2.7 Validation of Network Inference Using GO Enrichment and ROC Analysis

To evaluate the biological relevance of network inference results, Gene Ontology (GO) enrichment analyses were performed on ranked gene lists generated from RWR outputs. Gene subsets were selected at multiple thresholds, ranging from 50 to 800 in increments of 50. Biological Process enrichment from these subsets was determined using the enrichGO function from the clusterProfiler package (Wu et al. 2021), with mouse annotations from org.Mm.eg.db. A series of false discovery rate cutoffs (from 0.0001 to 0.1, including the commonly used 0.05) and enrichment score thresholds were systematically applied. For each gene set, enriched GO terms were filtered based on the specified cutoffs and compared against RNA‐seq‐derived GO terms to assess overlap. Receiver operating characteristic (ROC) curves were generated by assigning positive labels to terms recovered by both RWR and RNA‐seq and calculating the area under the ROC curve (AUC) using the pROC package. The performance was further evaluated using positive predictive value (PPV) and negative predictive value (NPV) metrics.

To visualize consistency and sensitivity of GO term recovery, Venn diagrams were created comparing *in silico* predictions and RNA‐seq results. When analyzing overlapping pathways between the *in silico* predictions and RNA‐seq results, we noted a substantial portion of pathways identified only by the network model had similar functional capacity compared to the overlapping pathways. To ensure that these pathways were not excluded due to nuances in pathway naming conventions, we performed semantic similarity analysis between the overlapping pathways and the pathways only identified *in silico* using the GOSemSim package (Yu et al. 2010). We then applied a global threshold of 0.7 across the similarity matrix and appended pathways from the *in silico* model to the overlapping group if they met this threshold. Lastly, to intuitively explore the relationship between cutoff thresholds and prediction quality, a 3D surface map of mean PPV values across gene subset sizes and FDR combinations was generated using plotly, following Gaussian smoothing of the raw metric matrix. This visualization enabled a comprehensive assessment of enrichment robustness across parameter settings.

### 2.8 Validation of GO Enrichment Using Immunohistochemistry

To validate the age‐dependent influence of IFP secreted factors on mitochondrial activity, as predicted via network perturbation and transcriptomic analyses, we performed immunohistochemistry staining for succinate dehydrogenase complex subunit A (SDHA) on chondrocytes exposed to IFP‐conditioned media. SDHA is an essential component of oxidative metabolism and, as such, positive immunostaining represents mitochondrial abundance and metabolic capacity of the tissue. Briefly, chondrocytes were isolated from aged male mice using previously established protocols (Gosset et al. 2008). Isolated chondrocytes were cultured for 1–2 weeks and then treated with media conditioned with either young or aged IFPs for 72 h.

Following treatment, chondrocytes were fixed with 4% paraformaldehyde in PBS for 5 min. Plates were then washed with PBS thrice for 3 min per wash. Chondrocytes were permeabilized with 0.1% triton‐X in PBS for 15 min, blocked using blocking buffer (3% bovine serum albumin, 5% goat serum) for 1 h, and incubated overnight at 4°C in primary antibody solution (1:250 SDHA in blocking buffer; Proteintech, Cat no. 14865‐1‐AP). The following day, plates were washed and incubated in a secondary antibody solution (1:400 Alexa Fluor 488 goat anti‐rabbit IgG in blocking buffer; Invitrogen, Cat no. A11008) for 1 h at room temperature, protected from light. Plates were then washed and stained with DAPI (1:500 in PBS) for 2 min. Stained chondrocytes were kept in PBS, protected from light, at 4°C until imaging. Chondrocytes were imaged using a Nikon AX/AXR confocal microscope at 20 × magnification. Laser power and gain were consistent across all samples. Five images were taken per well at random locations by an investigator masked to treatment allocation. SDHA fluorescence intensity was analyzed using Nikon Elements General Analysis 3.

## 3 Results

### 3.1 Validation of Cartilage Transcriptomic Dataset as a Foundation for Network Modeling

**FIGURE 1:**
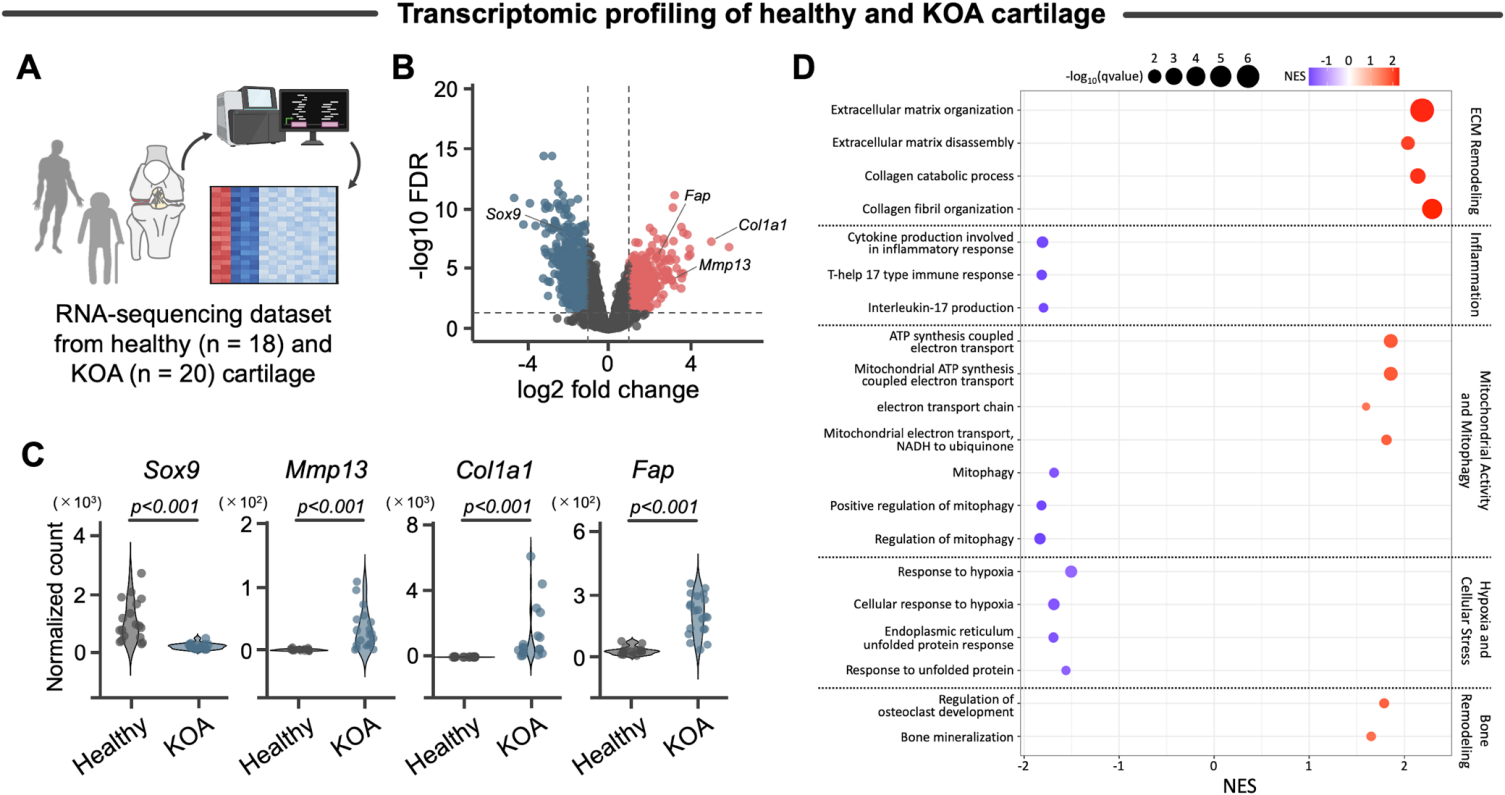
Validation of aging‐associated transcriptomic data as a biologically relevant foundation for network‐based modeling. (A) Overview of the transcriptomic dataset used in this study (GSE114007), which includes articular cartilage samples from healthy adults (*n* = 18; mean age: 38 years) and individuals diagnosed with KOA (*n* = 20; mean age: 66 years). This dataset served as the foundation for network construction, enabling the identification of age‐ and disease‐associated transcriptional changes in cartilage tissue. (B) Differential gene expression analysis comparing healthy and KOA cartilage samples. A volcano plot summarizes the analysis results, highlighting significantly differentially expressed genes (FDR < 0.05 and |log2 fold change| > 1). In total, 1294 genes were identified as differentially expressed, indicating widespread transcriptional reprogramming associated with KOA. (C) Expression profiles of representative cartilage‐related genes. Sox9, a key transcription factor essential for chondrogenesis, was significantly downregulated in KOA cartilage. Conversely, genes associated with catabolic or fibrocartilaginous remodeling, including Mmp13, Col1a1, and Fap, were upregulated. (D) Dot plot summarizing select Gene Ontology Biological Processes of GSE114007 that are associated with classical features of osteoarthritis. Positive NES represents positive enrichment in KOA samples. FDR, false discovery rate; KOA, knee osteoarthritis; NES, normalized enrichment score.

To establish a foundational network model for aging‐associated signaling in cartilage, we sought to construct a data‐driven network specific to cartilage tissue. For this, we first accessed transcriptomic data from GSE114007, a publicly available dataset of gene expression profiles of articular cartilage from the knee joints of healthy adults and people with KOA (Figure 1A) (Fisch et al. 2018). We processed and normalized raw count data using TMM normalization after filtering lowly expressed genes, resulting in expression profiles for 13,629 genes.

As a preliminary validation of the dataset, we performed differential expression analysis between healthy and KOA cartilage. This analysis identified 1294 differentially expressed genes (false discovery rate < 0.05 and |log2 fold change| > 1), of which 800 were upregulated and 494 were downregulated in KOA cartilage (Figure 1B). Notably, *Sox9*, a key chondrogenic transcription factor (Akiyama et al. 2002), was significantly downregulated, while *Mmp13*, a major catabolic enzyme implicated in cartilage degradation (Mitchell et al. 1996), was upregulated in KOA cartilage. In addition, *Col1a1*, a fibrocartilage marker (Housmans et al. 2022), and *Fap*, a fibroblast activation protein involved in tissue remodeling (Jacob et al. 2012), were also upregulated (Figure 1C). Gene set enrichment analysis of the dataset further demonstrated that the transcriptional profile of KOA cartilage captures disease‐associated biological processes, including extracellular matrix remodeling, shifts in inflammatory profiles, and metabolic reprogramming (Figure 1D and File S1). These findings are consistent with well‐established clinical features of KOA, including the loss of chondrogenesis and a shift toward a fibrocartilaginous, tissue‐degenerative phenotype (Tang et al. 2025). Together, these results support the use of GSE114007 as a representative dataset for modeling transcriptional chondrocyte responses in the context of KOA.

### 3.2 Construction of a Physiologically Relevant Network to Model Age‐Related Signaling in Cartilage

**FIGURE 2:**
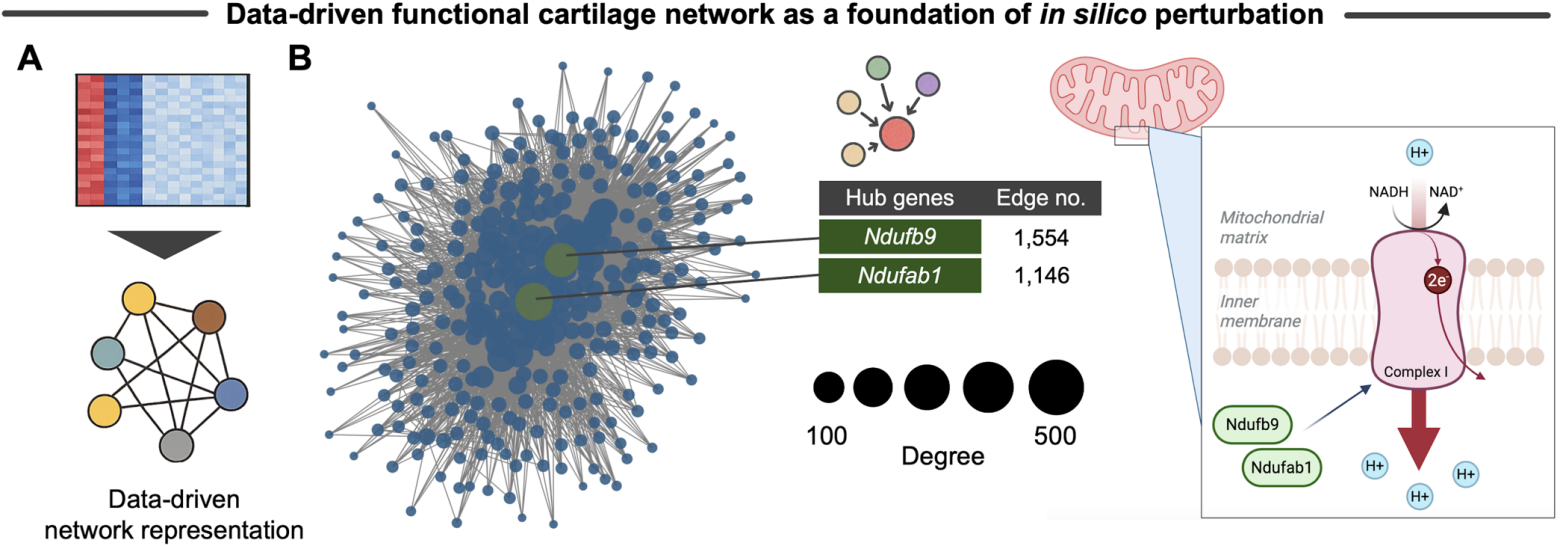
Construction of a cartilage‐specific transcriptional network from aging‐associated transcriptomic data. (A) Construction of a gene co‐expression network using WGCNA. This systems biology approach grouped genes into modules based on shared expression patterns across samples. From this analysis, we computed the TOM and retained the top 50,000 gene–gene interactions to construct a biologically meaningful and interpretable co‐expression network specific to cartilage. (B) Analysis of network topology and identification of hub genes. Nodes with high centrality were considered network hubs, representing genes with extensive co‐expression connections. Among these, mitochondrial complex I components Ndufb9 and Ndufab1 emerged as prominent hub genes. TOM, topological overlap matrix; WGCNA, Weighted Gene Co‐expression Network Analysis.

Building upon this validated dataset, we next aimed to construct a cartilage‐specific transcriptional network that provided systems‐level insights into aging‐associated signaling (Rintala et al. 2022). We employed WGCNA, a widely used systems biology method that groups genes into modules based on co‐expression patterns (Langfelder and Horvath 2008). By computing the TOM, which quantifies not only the direct correlation between gene pairs but also their shared network neighbors, we constructed a robust co‐expression network that reflects underlying biological organization (Figure 2A). To ensure network interpretability and manageability, we retained the top 50,000 weighted interactions based on TOM similarity scores and constructed a directed graph to model the resulting gene–gene interaction network.

The resulting network exhibited a scale‐free, hub‐centered architecture (Figure 2B), which is a key feature characteristic of biological system networks (Albert 2005). Centrality analysis revealed a subset of hub genes, identified as nodes with high connectivity and topological importance within the network. Notably, *Ndufb9* and *Ndufab1*, both core components of mitochondrial complex I involved in oxidative phosphorylation (Signes and Fernandez‐Vizarra 2018), emerged as top hub genes within the network (Figure 2B). Given that mitochondrial dysfunction has been implicated in cartilage aging and degeneration and directly disrupts cartilage homeostasis by altering collagen organization, crosslinking, and matrix stiffness (Loeser et al. 2016; Bubb et al. 2021), the prominence of these genes suggests that our network captures key features of mitochondrial contributions to cartilage structural integrity. For this reason, we used this network as the structural backbone for our subsequent analyses focused on receptor‐mediated downstream chondrocyte responses within the cartilage microenvironment.

### 3.3 In Silico Perturbation Predicted Chondrocyte Responses to Extracellular Aging Cues

**FIGURE 3:**
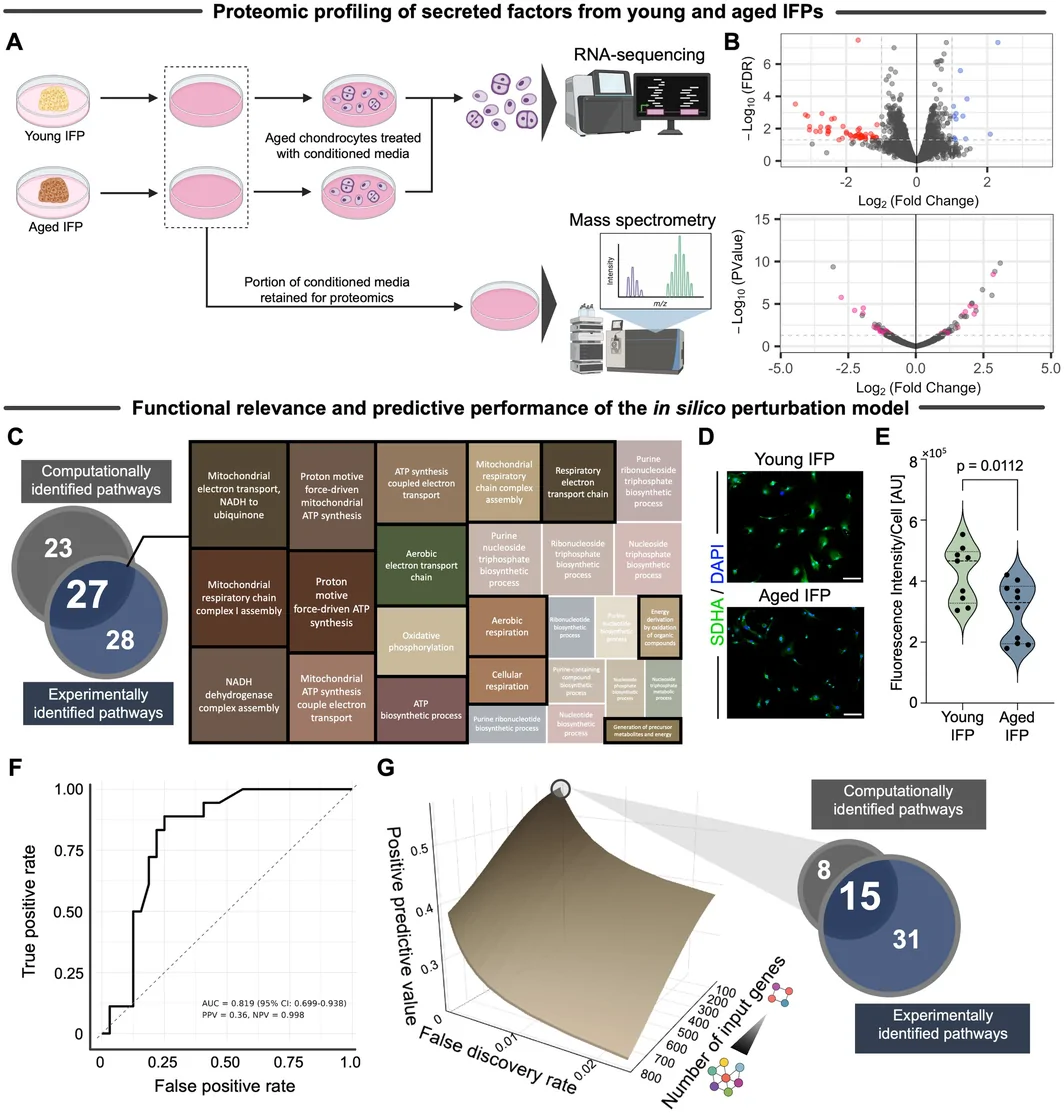
*In silico* perturbation predicts chondrocyte transcriptional responses to age‐associated extracellular cues. (A) Experimental design to validate the network‐based model. Aged primary chondrocytes were treated with conditioned media from young or aged IFPs, and transcriptomic responses were assessed by bulk RNA‐seq. (B) Volcano plots showing 84 differentially expressed genes (FDR < 0.05 and |log2 fold change| > 1) in aged chondrocytes exposed to aged versus young IFP‐conditioned media (top) and mass spectrometry‐based proteomic outputs (bottom) with secreted proteins used as RWR inputs highlighted in pink. (C) Venn diagram showing the number of significantly (FDR < 0.05) enriched GO biological function identified independently from RWR‐predicted gene sets and RNA‐seq, with 27 pathways considered overlapping. These shared pathways were visualized as a treemap, revealing predominant enrichment in mitochondrial‐related processes (outlined). Box sizes in the treemap are determined based on pathway enrichment score from RWR‐predicted GO outputs, with larger boxes indicating a greater enrichment score. (D) Representative images of SDHA immunostaining in aged chondrocytes cultured in media conditioned in the presence of either young or aged IFPs. SDHA (red) and DAPI (blue). Scale bar = 100 μm. (E) Average fluorescence intensity of SDHA immunostaining normalized to cell count. (F) ROC analysis comparing RWR predictions against RNA‐seq data. (G) Parameter optimization of the RWR model for improved prediction accuracy. We evaluated the effects of tuning two key parameters: The FDR threshold for GO enrichment and the number of RWR‐prioritized genes used as input. Applying more stringent FDR cutoffs increased PPV by enhancing enrichment specificity, while simultaneously narrowing the input gene set. FDR, false discovery rate; GO, gene ontology; IFP, infrapatellar fat pad; PPV, positive predictive value; RWR, Random Walk with Restart; SDHA, Succinate dehydrogenase complex subunit A.

To evaluate whether *in silico* receptor gene perturbation could reliably model transcriptional responses of aged chondrocytes, we compared the genes prioritized by RWR on a cartilage‐specific gene network with those experimentally identified via bulk RNA‐sequencing of aged primary chondrocytes treated in vitro with IFP‐conditioned medium derived from young and aged donors (Figure 3A). Among the top‐ranked genes prioritized by the RWR model were key regulators of mitochondrial function (NDUFB9 and NDUFAB1), ribosomal activity (RPS15, RPL32, and RPS16), and protein processing (CHCHD2, TOMM6, SPCS1, and LMTOR5). A full list of ranked genes prioritized by the model is included in File S2. As previously reported (D'Amico et al. 2025), differential expression analysis of cultured chondrocytes identified 84 differentially expressed genes (false discovery rate < 0.05 and |log2 fold change| > 1) upon exposure to aged IFP‐conditioned medium, of which 17 were upregulated and 67 were downregulated in aged chondrocytes (Figure 3B; top graph). We then identified differentially abundant proteins within media conditioned with young or aged IFPs. Given the exploratory nature of the network inference, we applied a nominal *p*‐value threshold (*p* < 0.05), rather than performing multiple testing correction (e.g., false discovery rate), to retain potentially relevant proteins for downstream model integration. A total of 30 proteins met this threshold, of which 10 were upregulated and 20 were downregulated in aged IFPs relative to young counterparts (Figure 3B; bottom graph).

We assessed the biological relevance of the top‐ranked genes from the RWR model and the differentially expressed genes identified by RNA‐seq using GO enrichment overlap analysis. As a representative scenario, we evaluated the RWR model using a commonly used gene set size (i.e., top 50 genes), combined with default enrichment parameters (false discovery rate = 0.05; fold enrichment = 0). The resulting GO enrichment analyses revealed 46 significantly enriched pathways from the RNA‐seq dataset and 50 pathways from the RWR‐based gene set. Among these, 18 pathways were shared between the two datasets. Noting that several pathways uniquely identified *in silico* appeared closely related to the overlapping pathways, we performed a semantic similarity analysis between the *in silico* and overlapping datasets. Pathways from the *in silico* set with a similarity score ≥ 0.70 with any of the overlapping pathways were subsequently reclassified as part of the overlapping group (Figure S1). This led to a total of 27 pathways considered overlapping between the *in silico* model and the experimental dataset, as illustrated by the Venn diagram (Figure 3C). A large portion of the remaining pathways identified only among the network model output were associated with translation, including *ribosome biogenesis, rRNA processing*, and *cytoplasmic translation* (Table S2). The presence of translation‐associated pathways unique to the *in silico* output may be an artifact of network structure, as there may be functional coupling between translational and mitochondrial processes (Chang et al. 2025). Thus, we expect genes encoding for translation‐related processes to be adjacent to or near mitochondrial genes—such as those identified as hub nodes in the network model—in weighted gene co‐expression networks. As such, RWR‐based perturbation of the network would preferentially highlight translation‐related pathways due to their proximity with known hub genes. Most pathways specific to the RNA‐seq dataset were associated with immune responses, such as *Type I interferon‐mediated signaling pathway* and *positive regulation of defense response* (Table S3). The inability of the model to capture these pathways reflects potential limitations of the mass spectrometry‐based proteomics approach used to identify in silico perturbation inputs, which may not be sufficient to identify small proteins and proteins present in low abundance (Altelaar et al. 2013; Ahrens et al. 2022), such as immune‐mediating factors. Despite these differences, the pathways identified by the network model and the experimental approach had a best match average semantic similarity score of 0.637, indicating moderately high functional similarity between the two datasets (Figure S2). Notably, a substantial portion of the overlapping pathways were associated with mitochondrial dysfunction, including *mitochondrial respiratory chain complex I assembly* and *cellular respiration* (Figure 3C and Table S4). We performed SDHA staining to confirm the functional relevance of these results. SDHA fluorescence intensity was significantly lower in aged chondrocytes exposed to secreted factors from aged IFPs compared to aged chondrocytes exposed to secreted factors from young IFPs (Figure 3D,E). These findings highlight a functional convergence between transcriptomic and network‐based approaches, supporting the biological validity of the computational model in capturing disease‐relevant mitochondrial processes.

To further evaluate the predictive performance of the *in silico* perturbation framework, we conducted ROC analysis using the RNA‐seq differential expression output as the reference. Under the same parameter settings, the model achieved an AUC of 0.819 (95% CI: 0.699–0.938) and a PPV of 36.0%, indicating moderate discriminatory power but limited precision (Figure 3F). The NPV remained high (99.8%), reflecting the model's robustness in excluding false positives under lenient filtering conditions.

Given the moderate PPV observed under default conditions, we next investigated how parameter tuning could improve model performance. The predictive accuracy of the RWR model proved sensitive to both enrichment thresholds and the scope of prioritized genes. Specifically, applying more stringent FDR cutoffs during pathway enrichment improved PPV, while simultaneously narrowing the input gene set (Figure 3G). We interpret these findings to suggest that PPV reflects a balance between statistical stringency and biologically‐guided gene selection (i.e., the number of genes considered for enrichment), and that focusing on genes near receptor seeds—likely representing functional partners—further increased agreement with true chondrocyte transcriptomic responses. Importantly, because predictive value was calculated relative to an independent, experimentally derived dataset, improved PPV under more stringent conditions indicates that the retained genes captured biologically relevant transcriptional programs rather than reflecting an artifact of network topology. This suggests that statistical filtering alone is insufficient, and prioritizing genes with a higher likelihood of biological relevance is equally critical. As such, optimizing both factors is necessary to refine the predictive power of *in silico* perturbation models. Together, these results demonstrate that age‐associated ligands derived from the IFP exert distinct and predictable effects on chondrocyte transcriptional programs, which can be faithfully captured using receptor‐centered random walk simulations on tissue‐specific gene networks. Furthermore, the capacity of the model to capture mitochondrial pathways identified experimentally highlights the high fidelity of such network‐based approaches at predicting hub gene responses to age‐associated extracellular cues.

## 4 Discussion

This study presents a computational framework that integrates tissue‐specific transcriptomic data, age‐associated extracellular proteomic inputs, and network‐based propagation modeling. We show that RWR‐based gene prioritization can be used to identify intracellular signaling modules most responsive to specific extracellular ligands. This approach not only helps predict which transcriptional programs may be activated, but it also facilitates the identification of key receptors that drive these intracellular responses. These insights could be further enhanced by combining RWR with an *in silico* gene deletion approach, in which the removal of individual receptors is simulated to infer how blocking specific ligand‐receptor interactions may alter the predicted intracellular signaling landscape. Such perturbation analysis provides insight into receptor‐specific signaling cascades and highlights potential regulatory nodes that mediate the molecular effects of extracellular stimuli on chondrocyte state.

To validate our computational framework, we used this model to elucidate how aging perturbs chondrocyte signaling in articular cartilage. By constructing a cartilage‐specific co‐expression network from human RNA‐seq data and integrating murine IFP secretome profiles, we simulated ligand‐triggered intracellular signaling using a RWR algorithm. This approach enabled us to predict transcriptional consequences of age‐altered extracellular cues *in silico* and revealed that mitochondrial dysfunction represents a central convergent pathway disrupted in aged cartilage. As mitochondria play a central metabolic and multifunctional role within articular cartilage (Kan et al. 2021), the convergence of mitochondrial pathways between computational perturbations and experimental conditions may reflect the high sensitivity of chondrocyte‐specific mitochondria to age‐related extracellular stressors. Importantly, our model not only recapitulated known transcriptomic responses observed in chondrocytes exposed to aged IFP‐conditioned medium but also offered mechanistic insights into receptor‐mediated signaling rewiring with aging. These findings demonstrate that *in silico* perturbation recapitulates physiologically relevant, age‐related signaling dynamics in cartilage and may be broadly applicable to other tissue systems undergoing age‐related degenerative changes.

Using cartilage‐IFP crosstalk as an example, our application of the RWR method to simulate signal propagation from extracellular ligands through a gene co‐expression network represents a novel approach for modeling paracrine signaling. Unlike conventional pathway enrichment methods, which are limited by predefined annotations, our diffusion‐based approach captures context‐specific interactions and permits the quantification of ligand‐specific influence across diverse biological modules. Compared to existing methods such as Cytotalk, which rely on cell–cell communication modeling from single‐cell transcriptomic data (Hu et al. 2021), our approach does not require cell‐type resolution and, instead, leverages tissue‐specific co‐expression topology to infer downstream signaling. Importantly, this framework moves beyond prior studies that have examined age‐related changes in joint tissues or secreted factors in isolation. Aging is a multifaceted process involving shifts in tissue composition and paracrine communication (Basisty et al. 2020; Iijima, Gilmer, et al. 2023), all of which converge to influence cellular function. By integrating age‐associated extracellular proteomic inputs with transcriptomic network dynamics, our approach provides mechanistic insights into how the aging joint microenvironment perturbs intracellular signaling pathways in chondrocytes. These extracellular cues, many of which originate from non‐cartilaginous tissues such as the IFP and synovium, can modulate key intracellular signaling cascades and transcriptional programs in chondrocytes, ultimately contributing to the pathogenesis of age‐related KOA (Iijima, Zhang, et al. 2023). This integrative model offers a powerful platform to explore the systems‐level consequences of aging on cartilage homeostasis and may inform therapeutic strategies targeting age‐driven tissue degeneration.

Notably, mitochondria‐related biological functions were consistently highlighted in both the *in silico* network analysis and the in vitro transcriptomic profiling. In particular, *Ndufb9* and *Ndufab1*, both components of mitochondrial complex I (Wu et al. 2016), emerged as hub nodes in the data‐driven gene regulatory network. This implies that the network topology may inherently prioritize genes involved in the mitochondrial respiratory chain, potentially due to their central connectivity or regulatory influence. While such structural features of the network could partially account for the prominence of these genes, their independent detection in vitro supports the notion that mitochondrial perturbation is a genuine and biologically meaningful response. Typically, mitochondrial damage and dysfunction are considered derivatives of cell‐intrinsic pathological changes with aging (Xu et al. 2025). However, because the network framework is designed to examine cellular responses to the extracellular environment, intrinsic aging mechanisms are largely outside of the scope of the model. Accordingly, our results suggest that extracellular factors may complement age‐related shifts in baseline cellular programs. This is further supported by SDHA immunostaining results, which suggest that aged chondrocytes exhibit decreased mitochondrial content and impaired mitochondrial function when exposed to the aged IFP secretome compared to the young IFP secretome. Given the fundamental role of mitochondria in energy metabolism and redox homeostasis (Willems et al. 2015; Youle and van der Bliek 2012), the convergence of *in silico* and in vitro findings suggests that mitochondrial complex I may act as a key integrator or effector of the cellular response to extracellular and aging‐related signals. These results underscore the necessity of integrating network architecture with experimental validation to disentangle modeling artifacts from biologically relevant signals.

From a clinical perspective, our ability to use *in silico* perturbation—computational simulation of how age‐related changes in extracellular ligands impact chondrocyte gene expression—represents a powerful tool for guiding therapeutic development. Aging tissues often secrete maladaptive signals that accelerate cartilage degradation and blunt natural repair mechanisms (Diekman and Loeser 2024). By modeling these complex signaling changes *in silico*, we can identify key molecular pathways, such as the pro‐inflammatory *Csf1* or anabolic *Igf1* pathways, that may be selectively targeted to restore balance. This computational approach also allows us to predict how various treatments—ranging from mechanical loading to stem cell‐derived factors—might reprogram intercellular communication within the aged joint microenvironment. Such predictions have the potential to provide a rational, systems‐level framework to design and optimize interventions that directly address the altered signaling networks driving age‐related cartilage degeneration. Ultimately, the integration of *in silico* perturbation with experimental and clinical data can accelerate the translation of molecular insights into effective therapies for joint aging and osteoarthritis.

While our approach provides a robust framework for prioritizing ligand‐receptor interactions in aging, several limitations warrant mention. First, RWR models signal diffusion based on topological proximity within a co‐expression network, implicitly assuming that genes with similar expression profiles are functionally related. However, co‐expression does not equate to causal regulation, and this method may fail to capture critical post‐transcriptional mechanisms, nonlinear interactions, or transient signaling events that do not manifest at the steady‐state transcript level. This may explain why several well‐established mediators of age‐related diseases, such as SASP‐ and inflammation‐associated factors, do not emerge from the network model. Similarly, key regulatory nodes, such as RNA‐binding proteins, post‐translational modifiers, or microRNA‐mediated controls, may be underrepresented in the inferred signaling cascades. Second, while our use of murine IFP‐derived secretome data provided a physiologically relevant ligand source, interspecies differences between murine ligands and human receptor networks may introduce translational discrepancies. To our knowledge, there is currently no publicly available proteomic atlas of human IFP samples. To improve the fidelity of the computational framework to human joint aging, future work should include proteomic profiling of human IFP samples as perturbation inputs. However, the high convergence between the *in silico* and in vitro experimental results suggests that interspecies discrepancies do not severely hinder the predictive function of the network model. Finally, the joint microenvironment is spatially complex, comprising distinct subpopulations of chondrocytes in a three‐dimensional architecture. Our current framework relies on bulk transcriptomic data without explicit spatial context. Incorporating spatial transcriptomics with cell–cell contact information would further enhance this computational pipeline, not only increasing the resolution and predictive power of the model but also expanding upon its applicability to more complex questions pertaining to tissue aging and regeneration.

Collectively, this study demonstrates the utility of network‐based perturbation modeling to identify and prioritize aging‐associated extracellular signals that modulate cartilage transcriptional states. Our integrative framework bridges proteomic and transcriptomic layers to uncover how topological positioning of receptors governs tissue responses to paracrine cues. These insights advance our mechanistic understanding of joint aging and provide a scalable approach to identify interventional targets in degenerative musculoskeletal diseases.

## Author Contributions

All authors made substantial contributions in the following areas: (1) conception and design of the study, acquisition of data, analysis and interpretation of data, drafting of the article; (2) final approval of the article version to be submitted; and (3) agreement to be personally accountable for the author's own contributions and to ensure that questions related to the accuracy are appropriately investigated, resolved, and the resolution documented in the literature. The specific contributions of the authors are as follows: conceptualization: H.I. Methodology: T.J.M., F.A., H.I. Software: T.J.M., H.I. Investigation: T.J.M., H.I. Resources: F.A., H.I. Data curation: T.J.M., H.I. Visualization: T.J.M., H.I. Funding acquisition: F.A., H.I. Project administration: F.A., H.I. Supervision: F.A., H.I. Writing – original draft: T.J.M., H.I. Writing – review and editing: T.J.M., F.A., H.I.

## Funding

This work was supported by the National Institutes of Health, R01AG089455, R01AG090356.

## Conflicts of Interest

The authors declare no conflicts of interest.

## Supporting information


**File S1:** Differentially regulated (*q* value < 0.05) biological processes between healthy and KOA samples. Output based on GO database. Positive NES indicates positive enrichment in KOA samples.
**File S2:** List of genes prioritized by the RWR model when top 50,000 gene–gene interactions are retrained.

**Table S1:** List of differentially abundant proteins identified within IFP‐conditioned media samples and their corresponding receptors defined via the NicheNet database.
**Table S2:** List of all pathways identified via network model outputs. Blue shading indicates pathways that were identified as being highly similar (semantic similarity score ≥ 0.7) to the overlapping pathways.
**Table S3:** List of all pathways identified via experimental results.
**Table S4:** List of all pathways identified via both network model outputs and experimental results, prior to semantic similarity analysis.
**Figure S1:** Heatmap showing semantic similarity scores of GO terms identified via the network model and the original set of overlapping GO terms. Raw values of semantic similarity between the two datasets are included in the matrix, in which values above 0.7 are highlighted in red. The pathways with at least one semantic score above 0.7 met criteria for reclassification as overlapping pathways in subsequent analyses.
**Figure S2:** Heatmap showing semantic similarity scores of all GO terms identified via the network model and experimental approach.

## Data Availability

RNA sequencing data are available under the GEO Accession GSE264710, and proteomics data are available in the MassIVE repository under identifier MSV000102265. All raw data are available upon reasonable request to the corresponding author.
