# Supplementary material for: Network Model to Predict Age‐Related Transcriptional Reprogramming
Source: Aging Cell. 2026 Aug 22;25(9):e70683. doi: 10.1111/acel.70683 (PMC13499505; doi:10.1111/acel.70683)
Supplement: Supplementary file 1 [file ACEL-25-e70683-s001.docx]

**Network model to predict age-related transcriptional reprogramming**

Tyler J. McNeill PhD, Fabrisia Ambrosio PhD MPT, Hirotaka Iijima PhD PT

Supplementary Materials:

- **Supplementary Table 1:** Differentially abundant proteins and corresponding receptors
- **Supplementary Table 2:** Pathways identified via network model
- **Supplementary Table 3:** Pathways identified in vitro
- **Supplementary Table 4:** Pathways identified both via network model and in vitro
- **Supplementary Figure 1:** Semantic similarity of overlapping pathways and pathways identified via network model
- **Supplementary Figure 2:** Semantic similarity of all pathways identified via network model and in vitro

**Supplemental Table 1:** List of differentially abundant proteins identified within IFP-conditioned media samples and their corresponding receptors defined via the NicheNet database.

| **Genes Encoding for Differentially Abundant Proteins** | **Corresponding Receptors in NicheNet Database** |
| --- | --- |
| SFN | No corresponding receptors |
| PPIA | No corresponding receptors |
| KRT17 | No corresponding receptors |
| CASQ1 | No corresponding receptors |
| ATP2A1 | No corresponding receptors |
| S100A16 | No corresponding receptors |
| H1-2 | No corresponding receptors |
| ACTN3 | No corresponding receptors |
| MYL12B | No corresponding receptors |
| KRT75 | No corresponding receptors |
| NFE2L1 | No corresponding receptors |
| SCP2 | No corresponding receptors |
| CA2 | No corresponding receptors |
| CA1 | No corresponding receptors |
| YWHAH | No corresponding receptors |
| GANAB | No corresponding receptors |
| ERP44 | No corresponding receptors |
| ACADM | No corresponding receptors |
| TST | No corresponding receptors |
| PLG | PLAT, F2R, ITGB1, F3, PLGRKT, ITGA9, F2RL1, PLAU |
| NID1 | ITGB1, ITGB3, COL13A1, PTPRF, ITGA3, ITGAV |
| MUG1 | No corresponding receptors |
| PZP | MMP2, MMP9 |
| APOA4 | LDLR, LRP1, LRP2 |
| AHSG | INSR |
| F2 | F2RL2, F8, F5, F2R, F2RL1, F2RL3, THBD, GP1BA, ITGA2B, GP9 |
| IGKV12-41 | No corresponding receptors |
| PHGDH | No corresponding receptors |
| HBB-B2 | No corresponding receptors |
| MFGE8 | ITGB3, ITGAV, PDGFRB, ITGB5 |

**Supplemental Table 2:** List of all pathways identified via network model outputs. Blue shading indicates pathways that were identified as being highly similar (semantic similarity score ≥ 0.7) to the overlapping pathways.

| **Gene Ontology ID** | **Pathway name** |
| --- | --- |
| GO:0002181 | Cytoplasmic translation |
| GO:0140236 | Translation at presynapse |
| GO:0140241 | Translation at synapse |
| GO:0140242 | Translation at postsynapse |
| GO:0015980 | Energy derivation by oxidation of organic compounds |
| GO:0022904 | Respiratory electron transport chain |
| GO:0022900 | Electron transport chain |
| GO:0006091 | Generation of precursor metabolites and energy |
| GO:0009152 | Purine ribonucleotide biosynthetic process |
| GO:0009260 | Ribonucleotide biosynthetic process |
| GO:0046390 | Ribose phosphate biosynthetic process |
| GO:0046034 | ATP metabolic process |
| GO:0006164 | Purine nucleotide biosynthetic process |
| GO:0042274 | Ribosomal small subunit biogenesis |
| GO:0009205 | Purine ribonucleoside triphosphate metabolic process |
| GO:0009199 | Obsolete ribonucleoside triphosphate metabolic process |
| GO:0009144 | Purine nucleoside triphosphate metabolic process |
| GO:0009165 | Nucleotide biosynthetic process |
| GO:0009141 | Nucleoside triphosphate metabolic process |
| GO:0006120 | Mitochondrial electron transport, NADH to ubiquinone |
| GO:0042254 | Ribosome biogenesis |
| GO:0006979 | Response to oxidative stress |
| GO:0061136 | Regulation of proteasomal protein catabolic process |
| GO:0009150 | Purine ribonucleotide metabolic process |
| GO:0022613 | Ribonucleoprotein complex biogenesis |
| GO:0006364 | rRNA processing |
| GO:0009259 | Ribonucleotide metabolic process |
| GO:1903050 | Regulation of proteolysis involved in protein catabolic process |
| GO:0019693 | Ribose phosphate metabolic process |
| GO:0034599 | Cellular response to oxidative stress |
| GO:0016072 | rRNA metabolic process |
| GO:0070972 | Protein localization to endoplasmic reticulum |

**Supplemental Table 3:** List of all pathways identified via experimental results.

| **Gene Ontology ID** | **Pathway name** |
| --- | --- |
| GO:0035456 | Response to interferon-beta |
| GO:0051607 | Defense response to virus |
| GO:0035458 | Cellular response to interferon-beta |
| GO:0032543 | Mitochondrial translation |
| GO:0140888 | Interferon-mediated signaling pathway |
| GO:0009615 | Response to virus |
| GO:0034340 | Response to type I interferon |
| GO:0140053 | Mitochondrial gene expression |
| GO:0060337 | Type I interferon-mediated signaling pathway |
| GO:0071357 | Cellular response to type I interferon |
| GO:0009617 | Response to bacterium |
| GO:0048525 | Negative regulation of viral process |
| GO:0034341 | Response to type II interferon |
| GO:0002832 | Negative regulation of response to biotic stimulus |
| GO:0141060 | Disruption of anatomical structure in another organism |
| GO:0045071 | Negative regulation of viral genome replication |
| GO:0031640 | Killing of cells of another organism |
| GO:0141061 | Disruption of cell in another organism |
| GO:0042742 | Defense response to bacterium |
| GO:0045824 | Negative regulation of innate immune response |
| GO:2000342 | Negative regulation of chemokine (C-X-C motif) ligand 2 production |
| GO:0031348 | Negative regulation of defense response |
| GO:0050792 | Regulation of viral process |
| GO:0060338 | Regulation of type I interferon-mediated signaling pathway |
| GO:0035455 | Response to interferon-alpha |
| GO:0071346 | Cellular response to type II interferon |
| GO:0031349 | Positive regulation of defense response |
| GO:0032606 | Type I interferon production |

**Supplemental Table 4:** List of all pathways identified via both network model outputs and experimental results, prior to semantic similarity analysis.

| **Gene Ontology ID** | **Pathway name** |
| --- | --- |
| GO:0006119 | Oxidative phosphorylation |
| GO:0009060 | Aerobic respiration |
| GO:0045333 | Cellular respiration |
| GO:0010257 | NADH dehydrogenase complex assembly |
| GO:0032981 | Mitochondrial respiratory chain complex I assembly |
| GO:0042776 | Proton motive force-driven mitochondrial ATP synthesis |
| GO:0015986 | Proton motive force-driven ATP synthesis |
| GO:0042775 | Mitochondrial ATP synthesis coupled electron transport |
| GO:0042773 | ATP synthesis coupled electron transport |
| GO:0006754 | ATP biosynthetic process |
| GO:0033108 | Mitochondrial respiratory chain complex assembly |
| GO:0009206 | Purine ribonucleoside triphosphate biosynthetic process |
| GO:0009145 | Purine nucleoside triphosphate biosynthetic process |
| GO:0009201 | Ribonucleoside triphosphate biosynthetic process |
| GO:0009142 | Nucleoside triphosphate biosynthetic process |
| GO:0019646 | Aerobic electron transport chain |
| GO:0072522 | Purine-containing compound biosynthetic process |
| GO:1901293 | Nucleoside phosphate biosynthetic process |

**Supplementary Figure 1**: Heatmap showing semantic similarity scores of GO terms identified via the network model and the original set of overlapping GO terms. Raw values of semantic similarity between the two datasets are included in the matrix, in which values above 0.7 are highlighted in red. The pathways with at least one semantic score above 0.7 met criteria for reclassification as overlapping pathways in subsequent analyses.

**Supplementary Figure 2**: Heatmap showing semantic similarity scores of all GO terms identified via the network model and experimental approach.
